# Bioengineered Mesenchymal Stem/Stromal Cells in Anti-Cancer Therapy: Current Trends and Future Prospects

**DOI:** 10.3390/biom14070734

**Published:** 2024-06-21

**Authors:** Jesús I. Gil-Chinchilla, Agustín G. Zapata, Jose M. Moraleda, David García-Bernal

**Affiliations:** 1 Hematopoietic Transplant and Cellular Therapy Unit, Instituto Murciano de Investigación Biosanitaria (IMIB) Pascual Parrilla, Virgen de la Arrixaca University Hospital, University of Murcia, 30120 Murcia, Spain; jesusisaias.gil@um.es; 2 Department of Cell Biology, Complutense University, 28040 Madrid, Spain; zapata@bio.ucm.es; 3 Department of Medicine, University of Murcia, 30120 Murcia, Spain; 4 Department of Biochemistry, Molecular Biology and Immunology, University of Murcia, 30120 Murcia, Spain

**Keywords:** mesenchymal stem/stromal cells, cell therapy, anti-cancer therapy, targeted therapy, therapeutic vehicles

## Abstract

Mesenchymal stem/stromal cells (MSCs) are one of the most widely used cell types in advanced therapies due to their therapeutic potential in the regulation of tissue repair and homeostasis, and immune modulation. However, their use in cancer therapy is controversial: they can inhibit cancer cell proliferation, but also potentially promote tumour growth by supporting angiogenesis, modulation of the immune milieu and increasing cancer stem cell invasiveness. This opposite behaviour highlights the need for careful and nuanced use of MSCs in cancer treatment. To optimize their anti-cancer effects, diverse strategies have bioengineered MSCs to enhance their tumour targeting and therapeutic properties or to deliver anti-cancer drugs. In this review, we highlight the advanced uses of MSCs in cancer therapy, particularly as carriers of targeted treatments due to their natural tumour-homing capabilities. We also discuss the potential of MSC-derived extracellular vesicles to improve the efficiency of drug or molecule delivery to cancer cells. Ongoing clinical trials are evaluating the therapeutic potential of these cells and setting the stage for future advances in MSC-based cancer treatment. It is critical to identify the broad and potent applications of bioengineered MSCs in solid tumour targeting and anti-cancer agent delivery to position them as effective therapeutics in the evolving field of cancer therapy.

## 1. Introduction

Mesenchymal stem/stromal cells (MSCs) are a multipotent adult stem cell heterogeneous population with the ability to both self-renew and differentiate into cells particularly belonging to mesoderm-derived tissues such as bone, adipose tissue and cartilage [1,2]. These cells were first discovered in bone marrow by Friedenstein et al. and were initially called colony forming units–fibroblasts (CFU-Fb) [3], but later they were found in many other adult and extraembryonic tissues including adipose tissue, umbilical cord, dental pulp and amniotic membrane [4,5,6]. 

The therapeutic potential of MSCs is enormous, particularly because of their ability to modulate immune responses, regulate tissue homeostasis through the secretion of paracrine trophic factors with pleiotropic effects, and maintain tissue health and structure [4,5,7,8,9]. These properties position MSCs as a cornerstone in the development of repairing cell therapies with ongoing clinical trials exploring their potential for the treatment of acute/chronic inflammatory and/or degenerative diseases, such as osteoporosis, osteoarthritis, graft-versus-host disease, systemic sclerosis and myocardial infarction [10,11,12].

**Figure 1:**
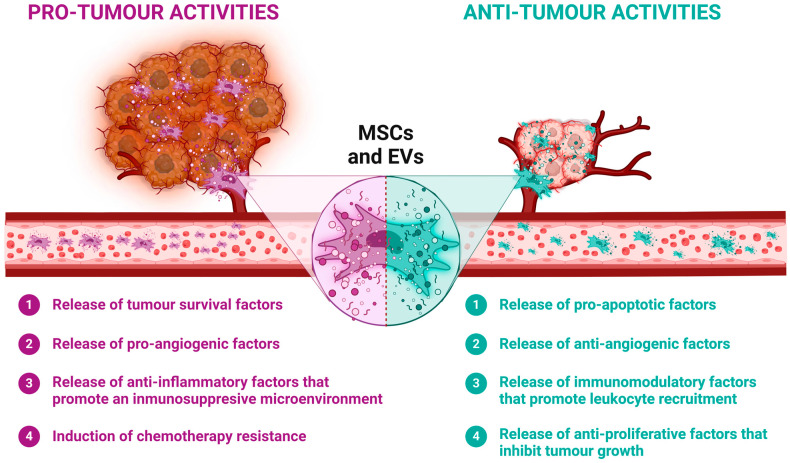
Dual roles of MSCs in tumour dynamics. MSCs possess unique properties that make them promising therapeutic agents. However, these same properties can also influence tumour development. MSCs can release a variety of factors that have both pro- and anti-tumour effects, influencing cellular tumour processes such as survival, proliferation, angiogenesis and chemotherapy resistance. These paracrine factors can be released directly into the tumour microenvironment or transported via extracellular vesicles (EVs). Created using BioRender.

However, the role of MSCs in cancer therapy presents a complex picture. While MSCs can home to tumour sites after their systemic administration, their impact on disease progression can be double-edged. On the one hand, they have been reported to directly fight some types of cancer by suppressing cancer cell proliferation through reducing the expression of positive regulators of the cell cycle [13,14,15,16]. On the other hand, it has also been found that MSCs may contribute to tumour growth by fostering a supporting tumour vascular network which modulates the peritumour immune environment, protecting it from immune attack, and promoting the proliferation and invasiveness of cancer stem cells [17,18,19,20,21] (Figure 1). This paradoxical behaviour of MSCs in tumours highlights the need for cautious and nuanced application in anti-cancer therapeutic approaches [22,23]. 

Therefore, the use of MSCs, particularly in cancer therapy, requires a delicate balance. Notwithstanding, several strategies are currently being developed to use MSCs as anti-cancer cell therapeutics. These include bioengineering MSCs to enhance their tumour homing and anti-tumour properties, or to carry as a “Trojan horse” some therapeutic agents that target and eliminate cancer cells more efficiently [24,25,26,27,28]. The development of such strategies requires a deep understanding of the interactions between MSCs, tumour microenvironment and immune system and calls for an interdisciplinary approach combining insights from cell biology, oncology and cell bioengineering to overcome current challenges and fully exploit the therapeutic potential of MSCs in cancer treatment.

## 2. Cutting-Edge Applications of Mesenchymal Stem/Stromal Cells in Cancer Therapy

### 2.1. Enhancing MSC Homing to Tumour Sites

Over the last few decades, the ability of MSCs to migrate into the tumour microenvironment has been extensively studied to analyse the effect they have on tumour biology. In preclinical models, systemically administered MSCs have been shown to be able to migrate and infiltrate inflamed or tumour tissues. At these tissues, local elements such as hypoxia, cytokines and chemokines stimulate the MSC infiltration and secretion of a variety of growth factors, thereby accelerating tissue repair [29]. In addition, tumours are able to recruit MSCs from distant tissues, such as adipose tissue or bone marrow, and direct their homing into the tumour microenvironment via inflammatory signals [30]. Several studies have already shown that MSCs are particularly recruited to different tumour types, such as breast cancer, liver cancer and glioma [31,32,33]. 

The tumour environment is rich in immune cells that, together with cancer cells, release a variety of soluble factors that influence the homing and infiltration of MSCs at sites of injury. Specific cytokines such as IL-6 attract MSCs to tumours, while other studies have reported similar recruitment driven by IL-8 in gliomas [34,35]. In addition, several growth factors have been found to promote MSC migration, including platelet-derived growth factor subunit B (PDGFB), vascular endothelial growth factor (VEGF) and transforming growth factor beta-1 (TGF-β1) [36]. The chemokine receptors CXCR4, CXCR6, CCR1, CCR7 and CCR9 also play an important role in the homing of MSCs to tumours [37]. Once MSCs reach the tumour, they contribute to its stroma by differentiating into fibrovascular cells such as endothelial cells, pericytes and possibly tumour-associated fibroblasts involved in the extracellular matrix remodelling [38]. However, further investigation into the molecular mechanisms that mediate specific migration to tumours may help to improve the efficacy of MSC-based therapeutics. 

Given their ability to target and integrate into malignant tissues, coupled with their immune-evasive properties, MSCs are considered an ideal approach for the delivery of anti-cancer therapeutics, potentially improving their efficacy compared to standard anti-cancer treatments [39,40]. 

Unfortunately, the homing efficiency of systemically administered MSCs is very low, with only a small fraction of these cells reaching their target tissues due to their entrapment in the lungs, liver and spleen vasculature, as demonstrated in several studies [41,42,43], limiting their expected therapeutic efficacy. To overcome this hurdle, the use of a variety of cell bioengineering strategies to improve MSC homing to inflamed and tumour tissues is being widely investigated. Several methodological approaches have been explored to alter the expression of different homing molecules on migrating MSCs, including priming with bioactive molecules, genetic engineering, enzymatic modifications or ligand conjugation techniques [44].

Exposure of MSCs to the pro-inflammatory cytokine TNF-α has been shown to upregulate CXCR4 expression, which may enhance the ability of MSCs to home to specific tissues, including tumours [45,46]. Additionally, priming MSCs with the cytokine TGF-β has been shown to increase their CXCR4-driven homing to glioblastoma, while MSCs stimulated with IL-1β showed an upregulated CXCR4 expression, increased production of metalloproteinases and enhanced migration [47,48]. Furthermore, pre-treating MSCs with valproic acid, erythropoietin, the iron chelator deferoxamine and granulocyte colony-stimulating factor (G-CSF) has been shown to improve their homing to inflamed tissues [49,50,51]. On the other hand, the peritumoural microenvironment that has a persistent inflammatory state, secreting various pro-inflammatory chemokines and cytokines (e.g., MCP-1, TGF-β, CXCL12, TNF-α and various interleukins), could prime MSCs enhancing their migration to tumours.

Another strategy to enhance MSC homing is the genetic modification to achieve permanent overexpression of key homing factors by viral transduction or alternatively transient overexpression by mRNA transfection. Zheng et al. introduced the CXCR4 gene into mouse bone marrow-derived MSCs by lentiviral transduction. Subsequently, mice with colitis-associated tumorigenesis that were injected with these CXCR4-overexpressing MSCs displayed a reduced tumour burden compared to mice treated with unmodified counterparts [52]. Overexpression of other chemokine receptors such as CXCR7 or the α4 chain of VLA-4 integrin, has demonstrated an improved MSC homing to inflamed/injured tissues [53,54]. Levy et al. aimed to enhance the ability of MSCs to tether and roll over endothelium by transfecting these cells with mRNA for PSGL-1 and sialyl Lewis X (sLeX), which are ligands for P- and E-/L-selectin, respectively [55], while Hervás-Salcedo et al. showed that the transient expression of CXCR4 by mRNA transfection improved MSC migration to inflamed tissues [56].

Enzymatic modification to transiently improve MSC homing has also been reported. Treatment of MSCs with an α(1,3)-fucosyltransferase VI or VII in the presence of GDP-fucose has been shown to convert CD44 into the E-selectin ligand HCELL glycovariant on the MSC surface. This modification by exofucosylation significantly enhances rolling contacts on E-selectin-expressing endothelial surfaces in the bone marrow microvasculature and inflamed tissues [57,58]. Notably, other studies have shown that exofucosylated MSCs exhibit enhanced migration towards specific pro-inflammatory chemokines such as CCL5, CCL20 and CXCL16 [59]. In addition, cell surface engineering techniques allow the direct conjugation of desired ligands, rather than modifying existing surface glycoproteins. For example, attaching sLeX to MSC surfaces via a biotin–streptavidin link, coupling E-selectin-targeting peptides to the MSC membrane, or conjugating recombinant CXCR4 to the phospholipid DMPE-PEG enhanced MSC rolling on P- and E-selectin-coated surfaces and migration to inflamed vascular endothelium in vivo [60,61,62,63].

### 2.2. MSC-Derived Extracellular Vesicles 

Extracellular vesicles (EVs) are membrane-derived nanostructures, including microvesicles, exosomes and apoptotic bodies (0.1–2 nm, 40–100 nm, and >1 μm in diameter, respectively) released by cells, including MSCs, under physiological and pathological conditions. Their internal contents depend on the cell of origin and may include proteins, enzymes, growth factors, carbohydrates, lipids and nucleic acids such as double-stranded DNA, mRNAs, long non-coding RNAs or microRNAs [64]. EVs have been shown to be involved in intercellular communication between MSCs and target cells, regulating the immune response and tissue repair [65]. Thus, MSC-derived EVs are considered a promising therapeutic alternative because they recapitulate the biological properties of MSCs themselves, reduce undesirable side effects such as toxicities due to MSC infusion and have potential for use in gene delivery, regenerative medicine and immunomodulation [66]. 

In the context of cancer, it has been suggested that MSC-derived EVs may facilitate the delivery of their cargo to tumour cells. However, like the MSCs from which they are derived, they can either suppress or promote tumour growth through different mechanisms including modulation of tumour angiogenesis, inhibition of cell proliferation, promotion of apoptosis, and facilitation of tumour growth and metastasis [67,68,69]. MSCs may influence tumour angiogenesis through mechanisms involving increased VEGF secretion, which activates the ERK1/2 pathway [68], or via the transfer of oncogenic miRNAs that affect various processes in tumour cells, such as inhibition of PTEN, inhibition of apoptosis or induction of macrophage type M2 polarization [70,71]. MSC-derived EVs have also been implicated in inducing chemoresistance, particularly in breast and gastric cancers, by transferring miRNAs that modulate distinct cellular signalling pathways and promote a chemotherapy-resisting dormant state [72,73].

On the other hand, some studies have shown that MSC-EVs may contain a diverse array of miRNAs, including but not limited to miR-31, miR-223, miR-205 and miR-21, all of which play critical roles in regulating tumour dormancy, a property of tumours to persist as a small number of undetectable cells following the surgical removal of the primary tumour [72,74]. For example, Wu and colleagues demonstrated the efficacy of EVs derived from human Wharton’s jelly-derived MSCs in halting bladder tumour cell proliferation through inducing G0/G1 phase arrest in a dose-dependent manner [75]. Subsequently, a recent in vitro study unveiled that EVs from bone marrow MSCs can reduce the proliferation, migration and metastatic invasion of osteosarcoma cells by delivery of miR-206, a well-known tumour suppressor [76]. Another study demonstrated the capability of MSC-derived EVs containing miRNA-100 to significantly suppress angiogenesis by modulating the mTOR/HIF-1α/VEGF signalling pathway in breast cancer-derived cells [77], while MSCs carrying miR-23b have been shown to not only reduce the growth and invasion of breast metastatic cancer cells, but also to reduce their sensitivity to the chemotherapy drug docetaxel [74]. Thus, MSC-derived EVs, including EVs derived from modified MSCs, are increasingly being explored for their potential in cancer therapy. These bioengineered EVs can be tailored to enhance their therapeutic efficacy, targeting capabilities and to carry specific therapeutic agents.

EVs can be engineered to carry chemotherapeutic agents and deliver them directly to the tumour site, potentially increasing the efficacy of treatments while reducing systemic toxicity. This targeted approach helps to manage and treat cancer more effectively by ensuring that the drugs are delivered specifically to the cancer cells, minimising the impact on healthy tissue. There are different methods for transferring the desired cargo inside the EVs. Pre-loading methods involve modifying parental cells to package therapeutic cargoes into EVs during their biogenesis. This can be achieved through genetic manipulation, leading to the overexpression of therapeutic molecules, or by incubating drugs with parental cells to produce drug-containing EVs [78]. While pre-loading ensures stable and intact EV membranes, it is time-consuming and has low efficiency. On the other hand, post-loading methods are performed after EV isolation, and the cargoes are encapsulated either passively or actively. Passive loading involves hydrophobic drugs attaching to the EV membrane, while active loading involves physically or chemically permeabilizing the EV membrane to incorporate hydrophilic drugs. Techniques include electroporation, sonication, freeze/thaw cycles and the use of chemical permeabilizers [79,80]. Each strategy has pros and cons, with careful consideration needed to prevent EV membrane damage or EV aggregation [81,82].

Using these manipulation strategies, it is also possible to load MSC-derived EVs with cytotoxic chemotherapeutic agents such as doxorubicin, paclitaxel or gemcitabine. Such EVs have been shown to inhibit cancer cell growth, induce apoptosis and suppress epithelial–mesenchymal transition in oral squamous cell carcinoma and cervical cancer [83,84]. Other approaches include vesicle loading with specific miRNAs, siRNAs, mRNAs, ncRNAs, proteins and peptides that have previously shown anti-tumour activity [69,85,86]. Some studies have reported the use of MSC-EVs to counteract chemoresistance in glioblastoma multiforme cells by delivering anti-miR-9, with promising results in reversing the chemoresistance of this tumour [87]. In addition, MSC-EVs loaded with miR-146b, miR124, miR-145 or miR-122 showed anti-tumour activity in malignant glioma and hepatocellular tumours, respectively [88,89,90]. MSC-EVs containing miR-124a and miR-15a have been shown to reduce cancer stem cell viability and growth in a glioma and multiple myeloma model, respectively [91,92]. Another innovative approach involves engineering MSCs to produce TRAIL-expressing EVs, molecules known to induce cancer cell apoptosis [93]. 

Despite their promising properties such as stability, target specificity and non-toxicity, challenges remain in the clinical application of MSC-derived EVs. Issues such as the need for large-scale production, standardized manufacturing methods and stringent clinical regulations are critical to maintaining the consistency and functionality of these biological carriers. Current research supports the notion that these EVs can naturally home to cancer sites, enhancing their potential as drug delivery systems. Future developments could include surface modifications to enhance delivery, such as enabling EVs to cross biological barriers, such as the brain–blood barrier, more efficiently and accumulate at tumour sites, thereby increasing the therapeutic impact of their cargo [94]. However, rigorous dosage and efficacy studies are essential to advance the clinical application of EVs and ensure their safe and effective use in cancer therapy.

### 2.3. MSCs as Therapeutic Cell Vehicles 

**Figure 2:**
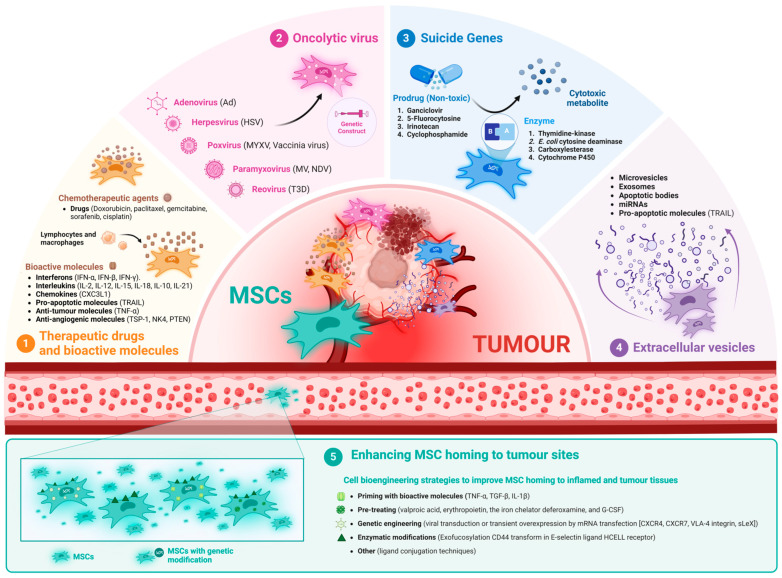
Different strategies to enhance the anti-tumour properties of both MSCs and MSC-derived EVs. (1) MSCs can be used to deliver chemotherapeutic drugs or bioactive molecules (e.g., interferons, interleukins, chemokines, and pro-apoptotic, anti-tumour or anti-angiogenic molecules) directly into the tumour microenvironment. (2) MSCs are effective oncolytic virus carriers because they can be easily infected, allowing viral replication and sustained viability until they reach the tumour microenvironment. (3) MSCs can be genetically engineered to carry suicide genes that encode specific enzymes that convert non-toxic prodrugs into cytotoxic metabolites directly in the tumour cells, increasing the specificity and efficacy of therapy while reducing systemic toxicity. (4) Bioengineered MSC-derived EVs can be customized to improve their therapeutic efficacy and capacity to deliver specific anti-cancer agents. (5) Various bioengineering strategies are being investigated to improve MSC homing to tumours, including priming with bioactive molecules, genetic engineering, enzymatic modification and ligand conjugation techniques. Created using BioRender.

There is considerable interest in using MSCs as carriers for tumour-targeted therapies because of their unique properties, particularly their natural affinity for homing tumours and to infiltrate the tumour environment [95,96,97,98]. Thus, MSCs have shown great promise in delivering drugs and genes in various tumours minimizing side effects of chemotherapeutic drugs and improving clinical outcomes. Therefore, the use of MSCs to directly deliver therapeutic agents to tumours has become a major focus of research. The therapeutic potential of MSCs originates from paracrine factors involving peptides, proteins and hormones, and the transfer of MSC-derived extracellular vesicles (EVs) containing different molecules inside. Remarkably, bioengineering strategies can prepare MSCs for the targeted delivery to tumours of various factors, focusing on a variety of biological approaches. For example, it has been shown that MSCs can be loaded with and subsequently release anti-cancer drugs [99,100,101,102]. However, the ability of MSCs to release a particular drug depends on both the MSC biology and the properties of these therapeutic compounds. Anti-cancer drugs loaded into MSCs have been found in various cellular components, affecting not only tumour cells but also gene expression and cellular functions of the MSCs themselves [101]. In addition, MSCs can be bioengineered to enhance their tumour-killing properties by preloading with suicide genes, oncolytic viruses, cytokines, anti-mitotic or anti-angiogenic factors, among others [98,103,104,105]. Accordingly, a first issue to develop is the analysis of the advanced techniques for bioengineering both MSCs and MSC-derived EVs to promote anti-tumour effects, which hold promise for the development of tumour-targeted therapies (Figure 2).

#### 2.3.1. Therapeutic Drugs and Bioactive Molecules

MSCs, with their inherent ability to take up a wide range of products including anti-cancer drugs from the culture medium, have emerged as a key tool in cancer therapy. Research has shown that human bone marrow-derived MSCs (hBM-MSCs) can be effectively primed with several chemotherapeutic agents, such as doxorubicin, paclitaxel, gemcitabine and sorafenib, through a simple incubation process [99,100,106,107,108]. This process facilitates the uptake of these drugs by the MSCs, albeit with varying degrees of efficiency. In particular, while hBM-MSCs show significant uptake of paclitaxel and other drugs, their interaction with pemetrexed is less effective, resulting in insufficient drug internalisation to adversely affect tumour cells [107]. Further investigation on MSCs from other sources, such as human adipose tissue (hAd-MSCs) or dental tissues, reveals a similar capacity to absorb several anti-cancer drugs, including cisplatin and paclitaxel [109,110,111]. This uptake is not uniform across MSC types, suggesting a dependence on the cellular properties specific to each MSC type. For example, the amount of paclitaxel absorbed by each hBM-MSC was quantified at approximately 2.7 pg/cell, whereas for other type of cells such as human olfactory bulb stem cells, it was 0.19 pg/cell, indicating a substantial capacity of uptake of MSCs that might involve specific transporters or membrane properties inherent to these cells [100].

The underlying drug uptake mechanisms by MSCs are diverse and include transporter-mediated entry, simple diffusion due to the lipophilic nature of some drugs, and various forms of endocytosis [112,113,114]. Specifically, the hydrophilic nucleoside analogue gemcitabine utilizes nucleoside transporters such as human concentrative nucleoside transporter 1 (hCNT1) and human equilibrative nucleoside transporter 1 (hENT1) for cell entry, suggesting that the efficacy of gemcitabine uptake may be closely linked to the expression levels of these transporters in MSCs [83,99,112]. On the other hand, lipophilic drugs such as paclitaxel can diffuse across the cell membrane, while larger molecules can be internalized via endocytotic pathways mediated by different receptors [111,114].

The therapeutic application of drug-loaded MSCs is two-fold, either through the utilization of MSC-conditioned media or by employing the MSCs as direct drug carriers. The conditioned media from drug-primed MSCs is particularly enriched with a secretome comprising various biologically active molecules, offering a targeted anticancer effect which exceeds that of the drugs alone. This enhanced effect is attributed to a more sophisticated drug release system that possibly influences MSC-EVs for improved drug delivery to cancer cells [115,116]. On the other hand, direct administration of drug-loaded MSCs facilitates an intimate cell-to-cell interaction, allowing a more direct and potent transfer of anti-cancer agents to the tumour cells. This method not only affects the MSC natural tumour-tropic properties but also provides a sustained release of the therapeutic agents, thereby maximizing the therapeutic impact while minimizing systemic side effects.

In vitro and in vivo studies have confirmed the efficacy of MSC-based drug delivery systems. Conditioned media derived from MSCs treated with gemcitabine or paclitaxel exhibit a significant inhibitory effect on the proliferation of several cancer cell lines, including some derived either from pancreatic adenocarcinoma and glioblastoma [100,117]. In addition, direct co-culture of cancer cells with drug-loaded MSCs has shown promising results in reducing tumour cell proliferation and attenuating tumour growth in some in vivo animal models [100,108,118].

In addition to their direct anti-tumour effects, drug-primed MSCs have shown potential for modulating tumour microenvironment-derived factors affecting angiogenesis and metastasis. For example, conditioned media from sorafenib-treated MSCs can inhibit endothelial cell proliferation, thereby affecting tumour vascularization [108]. In addition, the down-regulation of critical adhesion molecules by conditioned media from paclitaxel-loaded MSCs reduces the ability of tumour cells to metastasize [118], highlighting the multifaceted role of MSCs in cancer therapy. 

MSCs have also been genetically engineered to produce various bioactive molecules and immunomodulatory cytokines such as interferons (e.g., IFN-α, IFN-β, IFN-γ), interleukins (e.g., IL-2, IL-12, IL-15, IL-18, IL-10, IL-21), chemokines (e.g., CXC3L1), pro-apoptotic molecules (e.g., TRAIL), anti-angiogenic molecules (e.g., alpha-1 anti-trypsin (AAT), NK4, VEGFR1), and molecules with other anti-tumour properties (e.g., TNF-α), enhancing their ability to deliver specific therapeutic gene products, thereby reducing tumour growth, inducing apoptosis or acting as inhibitors of different pro-tumour factors [119,120,121,122,123,124,125,126,127,128,129,130,131,132]. It can be achieved by different methods: genetic modification of MSCs using viral vectors, as well as DNA plasmids or transposons. The choice of the appropriate method for genetic editing depends on the therapeutic goals and the specific targets involved.

Despite the efficacy of engineered MSCs in cancer therapy, their therapeutic outcomes as monotherapy in highly heterogeneous cancers remains limited, prompting the exploration of combined strategies to overcome chemotherapy resistance. For instance, the combination of TRAIL-engineered MSCs with temozolomide has shown greater efficacy in the treatment of glioblastoma than either treatment alone [133]. This synergistic effect that is attributed to the simultaneous induction of apoptosis and inhibition of cancer cell proliferation highlights the potential of engineered MSCs in the treatment of non-Hodgkin’s lymphoma [134]. However, concerns about the long-term safety of viral gene therapy have led to the consideration of non-viral gene transfection methods, despite their lower efficiency. Interferons, known for their anti-tumour properties, have been used in combination with tumour-specific antibodies (e.g., anti-PD-L1) or conventional chemotherapy such as β-cisplatin to control cancer progression in animal models [135,136,137]. Similarly, IL-12 has been recognized for its immunotherapeutic potential, stimulating T and NK cell activation and inhibiting tumour growth in both renal carcinoma and cervical tumour murine models [138,139]. In addition, Zhao et al. showed that MSCs transfected with a recombinant plasmid encoding IL-10 suppressed the proliferation of pancreatic cancer cells, reduced the growth of this xenografted tumour in vivo, and inhibited tumour angiogenesis [130]. MSCs overexpressing IL-21, a pro-inflammatory cytokine naturally produced by Th17 cells that inhibits regulatory T cell differentiation, effectively neutralized disseminated B-cell lymphoma [131]. Chen et al. found that MSC administration suppressed anti-tumour T cell responses and promoted tumour growth, while knocking down PD-L1 with shRNA prevented this effect [140]. Other strategies include the use of zoledronate-primed MSCs, which have a TGF-β-impaired secretion and induce Vδ2 T cell proliferation with anti-tumour properties [141]. These findings provide some explanations on the escape mechanisms that cancer exerts on the immune system. Bioengineered MSCs expressing proteins such as NK4, which inhibits hepatocyte growth factor (HGF), or thrombospondin-1 (TSP-1) variants, which suppress angiogenesis, have shown promising results in reducing tumour growth and vascularization in a lung metastatic and a glioblastoma tumour model, respectively [126,142,143]. However, the safety of such therapies, particularly the risk of teratoma formation, needs to be further evaluated. The bioengineering of MSCs to express other tumour suppressor proteins, such as bone morphogenetic protein 4 (BMP4), or phosphatidylinositol 3,4,5-trisphosphate 3-phosphatase (PTEN), has also explored and further demonstrated the versatility of MSCs in cancer therapy, opening new ways for the development of more effective and targeted cancer treatments [144,145,146].

The anti-neoplastic effects of modified MSCs with different anti-cancer drugs or bioactive/immunomodulatory molecules and the main mechanisms of action in tumour cells are summarized in Table 1.

#### 2.3.2. Oncolytic Viruses 

Oncolytic viruses (OV) are attenuated, non-pathogenic viruses designed to selectively recognize, infect and destroy tumour cells without affecting the rest of the healthy cell types where they are unable to replicate [105,119,147]. Its therapeutic use began at the end of the 19th century, with some controversy due to the poor clinical outcomes observed in early cancer patient trials. However, it was not until the end of the 20th century that oncolytic virotherapy was revived, due to the development of molecular biology and the introduction of genetic modification techniques, which not only allowed viruses with greater affinity for tumour cells to be isolated, but also modified or assembled to enhance their anti-tumour properties [119,148,149,150].

Most viruses used in oncolytic virotherapy, whether single- or double-stranded DNA or RNA, are usually based on human pathogens such as adenovirus (Ad), herpes simplex virus (HSV), measles virus (MV), coxsackievirus, vaccinia virus or reovirus, among others. However, viruses from other animal species, such as Newcastle virus, vesicular stomatitis virus or retroviruses, can also be used, and more than 10 viral families with their serotypes and subgroups have been studied as anti-tumour therapeutic agents [149,150,151,152]. Their mechanisms of action are dual, based on: (1) the selective destruction of tumour cells after infection (direct cellular oncolysis); and (2) the stimulation of the patient systemic anti-tumour immunity induced by the release of new viral particles generated by the lysis of tumour cells, which create a more pro-inflammatory tumour microenvironment that favours the immune attack and limits the evasion capacity of tumour cells (indirect cellular oncolysis) [105,119,147,148,151,153]. 

Unfortunately, these anti-cancer effects associated with oncolytic virus may be reduced when administered systemically intravenously, resulting in more occasional and transient responses than when administered locally (intratumorally) [154]. The main factors associated with this low anti-tumour efficacy after intravenous administration are (1) the ability of the patient immune system to recognize and neutralize the oncolytic viruses before they can reach the tumour and exert their therapeutic effects; (2) the presence of an inadequate tropism, whereby these oncolytic viruses are directed to tissues where they can be trapped or retained, such as the liver or spleen; (3) the presence of factors associated with the tumour development; and (4) the occurrence of factors associated with the tumour microenvironment that prevent their pre-penetration and intratumoural diffusion, including the release of chemokines and cytokines such as IL-10 and transforming growth factor β (TGF-β), among others [105,119,147,148,150,154].

Therefore, in recent years, new strategies have been developed to overcome these limitations: (1) increase of the tumour tropism (selection of entry receptors that are highly expressed by tumours); (2) improve their safety (restriction of viral replication to tumour cells only); (3) increase their therapeutic efficacy by inserting therapeutic transgenes that co-express cytokines and other molecules with anti-tumour activity; and (4) improve their biodistribution in the tumour environment [149].

Among the strategies to improve the biodistribution of oncolytic virus to tumour cells, different cell types (lymphocytes, myeloid cells, mesenchymal stem/stromal cells, etc.) as carriers of these oncolytic viruses to the tumour environment have been tested [150,152]. Of all these, MSCs have attracted most interest as oncolytic carriers due to (1) their easy capacity to be infected by oncolytic viruses, which allows them to replicate and produce new virions, remaining viable long enough to reach the tumour microenvironment; (2) their capacity for selective migration, achieving a more direct transport towards the tumour microenvironment (tumour tropism), where a large amount of pro-inflammatory cytokines and chemokines are released, favouring the chemoattraction of these MSCs carriers; and (3) their immunomodulatory properties, which prevent the recognition of these viruses by the patient innate and adaptive immune system [105,119,150,151,152,154,155,156].

The main preclinical studies evaluating the use of human MSCs as oncolytic virus vectors are summarized in Table 2.

#### 2.3.3. Suicide Genes

Gene-directed enzyme prodrug therapy, also known as suicide gene therapy, is a promising alternative of cancer treatment that goes far beyond the limits of conventional chemotherapy. This innovative therapy modality involves delivering a gene construct into the therapeutic cells encoding an enzyme that is capable of converting a non-toxic prodrug into a cytotoxic metabolite subsequently within the tumour environment, thereby concentrating the cytotoxic effect onto the malignant cells while minimizing the effect on healthy cells [214].

The most common example of this approach is the combination of the thymidine kinase (TK) gene from herpes simplex virus (HSV) with ganciclovir (GCV) as a prodrug. TK catalyses the phosphorylation of deoxythymidine and a wide range of nucleotide analogues, including GCV, which is then further phosphorylated by cellular kinases to its active and cytotoxic triphosphate form, GCV-TP [215]. Then, GCV-TP is incorporated into DNA during replication, ultimately leading to premature chain termination, and eventually inducing apoptotic cell death [216]. Although GCV-TP is not able to diffuse passively to adjacent cells, the gap junctions established between MSCs encoding the HSV-TK gene and the surrounding tumour cells allow the entry of GCV-TP into the tumour cells, hence causing cell apoptosis. This mechanism of action has been termed “bystander effect”. Additionally, the bystander effect can be augmented by cells that do not express the TK gene through the phagocytosis of apoptotic vesicles that contain GCV-TP. For example, MSCs expressing the HSV-TK gene in combination with GCV have provided promising results in the treatment of a variety of tumours, including colorectal cancer, melanoma, glioblastoma and breast cancer [217,218,219,220].

Another approach of suicide gene therapy is based on the use of the *Escherichia coli* cytosine deaminase (CD) gene in combination with the prodrug 5-fluorocytosine (5-FC). In this setting, therapeutic MSCs are bioengineered to express the CD enzyme, which converts 5-FC into 5-fluorouracil (5-FU), a potent cytotoxic metabolite for the neighbouring tumour cells [221,222]. Other examples include the combinations of the carboxylesterase gene with irinotecan, and the cytochrome P450 gene with cyclophosphamide. These combined treatments have been successfully tested in preclinical animal models of osteosarcoma, melanoma, and brain, breast and colorectal tumours [223,224,225,226,227].

The use of MSCs as delivery vectors for the suicide genes has shown remarkable progress in the targeted transfer of suicide genes. MSCs, known for their tumour-homing capabilities, can be genetically engineered to express the suicide gene, thereby directly delivering the gene to the tumour site. This strategy increases the specificity and efficacy of the therapy and reduces the risk of systemic toxicity. Moreover, some studies have explored the combination of suicide gene therapy with other therapeutic strategies to enhance its efficacy. For example, combining HSV-TK/GCV with immune checkpoint inhibitors, radiation or other targeted therapies (e.g., histone deacetylase inhibitors and valproic acid) can synergize to produce more robust anti-tumour responses, potentially overcoming resistance mechanisms and improving treatment outcomes [228,229,230].

Preclinical cancer models using MSCs as therapeutic vehicles of suicide genes are summarized in Table 3.

## 3. Clinical Trials

Among the changing scenarios in cancer therapy, MSCs have emerged as a promising tool, not only because of their regenerative capabilities, but also because of their abovementioned potential utility as delivery vehicles for anticancer agents. The use of MSCs with oncolytic viruses, genetically modified and engineered cells, and other therapeutic modalities is opening new avenues in the fight against various malignancies, as evidenced by completed and ongoing Phase I and II clinical trials (Table 4). 

Initial clinical attempts to fight different types of tumours combined MSCs with oncolytic viruses. For example, a Phase I/II clinical trial studied the systemic administration of autologous hBM-MSCs infected with the oncolytic adenovirus ICOVIR-5 (CELYVIR) for the treatment of paediatric and adult patients with solid metastatic and refractory tumours (NCT01844661). The authors found that CELYVIR had a very low systemic toxicity and a great safety profile and beneficial anti-tumour effects [103,232]. The same strategy but using allogeneic MSCs infected with ICOVIR-5 (AloCELYVIR), is being investigated alone or in combination with radiotherapy, to assess its safety, tolerability and preliminary efficacy for the treatment of diffuse intrinsic pontine glioma and medulloblastoma (NCT04758533). Another clinical study, identified as NCT03896568, which is still recruiting patients, involves the intra-arterial administration of allogeneic hBM-MSCs loaded with the oncolytic virus DNX-2401, also known as Delta-24-RGD. This treatment is being investigated in patients with recurrent glioblastoma, gliosarcoma and astrocytoma, while the Phase I/II clinical trial NCT02068794 is still evaluating the therapeutic efficacy of intraperitoneal administration of MSCs infected with oncolytic measles virus encoding thyroidal sodium iodide symporter (MV-NIS) in the treatment of ovarian, primary peritoneal or fallopian tube cancer. 

Subsequent studies have broadened the scope of bioengineered MSC applications. For example, the phase I/II TREAT-ME1 clinical trial evaluated the safety and efficacy of autologous MSCs delivering the gene HSV-TK (MSC_apceth_101) in combination with GCV, showing acceptable safety, tolerability and some signs of effectiveness in reducing tumour growth and metastases in advanced gastrointestinal adenocarcinoma (NCT02008539) [233]. 

The ability of MSCs to release therapeutic drugs or bioactive molecules into the tumour microenvironment has also been explored in several clinical trials. Thus, the TACTICAL trial is evaluating the combination of allogeneic MSCs overexpressing the TRAIL gene and chemotherapy in patients with metastatic lung adenocarcinoma to determine tolerability and efficacy (NCT03298763), while IFN-β or IL-12-producing MSCs are being evaluated for ovarian cancer or head and neck cancer treatment, respectively (NCT02530047 and NCT02079324). 

Furthermore, the clinical use of MSC-EVs for cancer treatment is currently one of the future challenges for cell-free therapies. An example of this approach is an active Phase I clinical trial to evaluate the therapeutic effects of MSC-EVs loaded with small interfering RNA (siRNA) against KrasG12D (iExosomes) in metastatic pancreatic ductal adenocarcinoma patients with KrasG12D mutation (NCT03608631). MSC-EVs are considered as a promising platform through which to further enhance the anti-cancer effects of traditional therapies, and of course more translational studies will be conducted in the foreseeable future to further reveal their therapeutic potential.

## 4. Challenges and Future Prospects 

Mesenchymal stem/stromal cells are widely used to treat various inflammatory and degenerative diseases due to their ability to engraft and repair damaged tissues, differentiate into different cell types and secrete a variety of soluble mediators with pleiotropic effects. However, the use of MSCs in the treatment of cancer must be approached with caution to minimize their potential to support tumour growth while maximizing their anti-tumour effects. Developing effective strategies involve genetically modifying MSCs or extracellular vesicles to enhance their anti-cancer effects or to deliver therapeutic agents to cancer cells as “Trojan horses”. Future understanding of the complex interactions between MSCs, the immune system and the tumour environment is essential for these advances. While challenging, these strategies aimed at enhancing MSC homing and engraftment in tumours following systemic administration, hold promise for fully unlocking the therapeutic potential of MSCs in precise and tailored anti-tumour therapies. Continued preclinical and clinical research is essential to develop safe and effective MSC-based cancer therapies that will ultimately improve the survival and quality of life of patients suffering from a wide range of malignancies.

## Figures and Tables

**Table 1:** Anti-tumour mechanisms of drug/biomolecule-loaded MSCs.

Loaded Drug/Molecule	Target Cancer	Therapeutic Effect	References
Doxorubicin	Lung melanoma metastases	Reduction of tumour cell viability	[106]
	Oral squamous cell carcinoma	Inhibition of tumour cell growth	[83]
Paclitaxel	Multiple myeloma	Inhibition of tumour cell growth	[99]
	Prostate, malignant glioma and melanoma cancer cell lines	Inhibition of tumour cell growth	[100]
	Oral squamous cell carcinoma	Inhibition of tumour cell growth	[83]
Gemcitabine	Oral squamous cell carcinoma	Inhibition of tumour cell growth	[83]
	Pancreatic carcinoma	Inhibition of tumour cell growth	[117]
Sorafenib	Glioblastoma multiforme	Inhibition of tumour cell growth and angiogenesis	[108]
Cisplatin	Mesothelioma and glioblastoma multiforme	Inhibition of tumour cell growth	[109]
IL-18, IFN-β	Intracranial glioma	Inhibition of tumour cell growth	[120,137]
IL-2	Glioma	Increased anti-tumour effects	[122]
IL-12, IL-15	Melanoma, lung cancer, pancreatic cancer, intracranial glioma and hepatoma	Direct anti-tumour effect and activation of cytotoxic T and NK cells	[123,124,125]
IL-10	Pancreatic cancer	Inhibition of tumour cell proliferation and angiogenesis	[130]
NK4	Pancreatic cancer	Inhibition of tumour cell proliferation and migration	[126,142]
TRAIL+/− temozolomide	Malignant glioma	Direct anti-tumour effect and apoptosis of tumour cells	[127,133]
TSP-1	Glioblastoma multiforme	Inhibition of tumour angiogenesis	[143]
BMP4	Malignant glioma	Increased anti-tumour effects	[144]
PTEN	Glioma	Induced cytotoxicity on tumour cells	[145,146]

**Table 2:** Preclinical studies using MSCs as carrier for oncolytic viruses.

Family	Natural Host	Type of OV	Host Cell	Target Cancer	Route	References
Adenoviridae	Human	Ad-ICOVIR5	AT-MSCs	Lung adenocarcinoma	IP	[157]
			AT-MSCs	Osteosarcoma	IP	[158]
			AT-MSCs	Lung adenocarcinoma	IT	[159]
		Ad-ICOVIR15-Ad.IC9	BM-MSCs	Lung cancer	IV	[160]
		Ad-ICOVIR15	Men-MSCs, BM-MSCs	Lung and pancreatic adenocarcinoma, melanoma	IP	[161]
			Men-MSCs + PBMNCs	Lung adenocarcinoma, epidermoid carcinoma, pharynx squamous cell carcinoma	IP	[162]
		Ad-ICOVIR15, Ad-ICOVIR15-cBITE	Men-MSCs	Lung adenocarcinoma and epidermoid carcinoma	IP	[163]
		Ad-ICOVIR15, Ad-ICOVIR17	AT-MSCs	Glioblastoma multiforme	IT, IV	[164]
		Ad-ICOCAV17	AT-MSCs	Osteosarcoma and brain tumours	IV	[165]
				Spontaneous lung carcinoma	IV	[166]
		Ad-hOC-E1	BM-MSCs	Renal carcinoma	IP	[167]
		Ad-RLX-PCDP	BM-MSCs	Pancreatic cancer	IV	[168]
		Ad-Ad5-HexPos3	BM-MSCs, AT-MSCs	Head and neck squamous cells carcinoma	IV, IP	[169]
		Ad-Ad5-Ki67/IL-15	Source of MSCs non-specified	Glioblastoma multiforme	IT	[170]
		Ad-5, Ad-3, Ad-5.Pk7-Delta24	BM-MSCs, AT-MSCs	Lung and breast tumours	IV	[171]
		Ad-AFPp-E1A, Ad-AFPp-E1A-122	WJ-MSCs	Hepatocellular carcinoma	IV	[172]
		Ad5/3-Δ19K-Luc-GFP, Ad5/3-TRAIL-GFP, Ad5/3- FCU1-GFP/5-FC	BM-MSCs	Pancreatic cancer	-	[173]
		Ad-Ad5/3 Ad-Ad5/3RGD-Luc	BM-MSCs	Ovarian carcinoma	IP	[174]
		Ad-Ad5/3-TRAIL	BM-MSCs	Pancreatic ductal adenocarcinoma	IV	[175]
		Ad-hTERTp-IL24	WJ-MSCs	Hepatocellular carcinoma	IV	[176]
		Ad-5-E3, Ad-WNTi	BM-MSCs	Hepatocellular carcinoma	IV	[177,178]
		Ad-CRAd-EGFP	BM-MSCs	Colon cancer	IV, IP	[17]
		Ad-CRAd, Ad-bic	MSCs-E1 (Gene Ad E1A/E1B)	Prostate cancer	IT	[179]
		Ad-CRAd, Ad5/3.CXCR4	BM-MSCs	Lung metastases of breast carcinoma	IV	[180]
		Ad-CRAdNTR (PS1217H6)	BM-MSCs	Colorectal cancer	IV	[181]
		Ad-CRAd5/F11	Men-MSCs	Colorectal cancer	IV, IP, IT	[182]
		AD-5/3-kBF5HRE-E1Awt	BM-MSCs	Melanoma, breast tumour	IO	[183]
		Ad-WT, Ad-RGD, Ad-5/3, Ad-CRAd	Source of MSCs non-specified	Gliomas (Glioblastoma multiforme)	IC	[184]
		Ad-WT, Ad-5-CRAd-S-pk7	BM-MSCs	Breast cancer	IT	[185]
		Ad-rAd.DCN Ad-rAd.Null	WJ-MSCs	Breast cancer lung metastatic	IV	[186]
		Ad5-Delta-24-RGD	Source of MSCs non-specified	Ovarian and breast cancer	IV	[187]
		Ad-Delta-24-RGD	BM-MSCs	Gliomas	IC, IA	[188,189,190]
		Ad5/35-Tet-on-E1b Pro-D24-ES-IL-24	UCB-MSCs	Gliomas	IV	[191]
		Ad-YSCH-01	DP-MSCs	Glossopharyngeal, bladder and breast squamous cancer	IV, IP, IT	[192]
Herpesviridae	Human	Herpes Simplex Virus (HSV)	Source of MSCs non-specified	Melanoma brain metastatic	IA	[193]
		HSV-R-LM249	BM-MSCs, AM-MSCs, AT-MSCs, DP-MSCs	Lung and brain metastases	IV	[194]
Poxviridae	Human and Bovine	Vaccinia virus (CAL1)	AT-MSCs	Colon cancer	IT	[195]
		Vaccinia virus (Copenhagen, Wyeth and LIVP strains)	AT-MSCs	Canine soft tissue sarcoma	IV	[196]
		Vaccinia virus (WT1/ACAM2000 and L14 strains)	AT-MSCs	Melanoma, lung carcinoma, myelogenous leukaemia	-	[197]
	Rabbit	MYXV-IL15	BM-MSCs	Pulmonary melanoma	IV	[198]
		MYXV-TNFSF14	AT-MSCs	Pancreatic adenocarcinoma	IP	[199,200]
		MYXV	AT-MSCs	Glioblastoma multiforme	IC	[201]
Paramyxoviridae	Human	MV	AT-MSC	Ovarian cancer	IP	[202,203]
			BM-MSC	Hepatocellular carcinoma	IV	[204]
				Lymphoblastic leukaemia	IV	[205]
	Birds	NDV (LaSota strain)	BM-MSC + Lactobacillus casei extract	Colorectal cancer	*-*	[206]
			BM-MSC	Human papillomavirus associated malignancy		[207]
		NDV (MTH-68/H)	BM-MSCs, AT-MSCs, WJ-MSCs	Glioblastoma multiforme	*-*	[208]
Reoviridae	Mammalians	Reovirus (T3D strain)	AT-MSC	Lung cancer	IV	[209]
			AT-MSC	Glioblastoma multiforme	*-*	[210]
			AT-MSC	Colorectal cancer	IT	[211]
			AT-MSC	Lung cancer	*-*	[212]
			WJ-MSC	Acute myeloid leukaemia	IV	[213]

Abbreviations: OV (oncolytic virus), Ad (adenovirus), MV (measles virus), MYXV (myxoma virus), NDV (Newcastle disease virus), RLX (relaxin), PCDP (biodegradable polymer), WNTi (Wnt-inhibiting decoy receptor), MSCs (mesenchymal stem/stromal cells), BM (bone marrow), AM (amniotic membrane), AT (adipose tissue), DP (dental pulp), WJ (Wharton’s jelly), Men (menstrual blood), UCB (umbilical cord blood), BITE (bispecific T cell engager), IT (intratumoural), IV (intravenous), IA (intra-arterial), IP (intraperitoneal), IC (intracranial), IO (intraocular), WT (wild-type), PBMNCs (peripheral blood mononuclear cells), DCN (decorin).

**Table 3:** Preclinical anti-tumour models using suicide genes expressing MSCs.

Suicide Gene/Prodrug	Tumour Preclinical Model	References
Thymidine kinase + ganciclovir	Melanoma lung metastasis	[217]
	Breast cancer Glioblastoma multiforme Colon cancer Malignant melanoma Intracranial glioma	[218] [219,231] [220] [228] [229]
*E. coli* cytosine deaminase + 5-Fluorocytosine	Glioblastoma multiforme Osteosarcoma	[221] [222]
	Colon cancer	[225]
Carboxylesterase + irinotecan	Glioma	[226]
Cytochrome P450 + cyclophosphamide	Colorectal and breast cancer	[227]

**Table 4:** Human MSC-based clinical trials targeting solid tumours.

Clinical Trial ID	Target Cancer	Therapeutic MSCs	Status	Location
NCT01844661	Solid metastatic and refractory tumours	Autologous MSC-ICOVIR-5 (CELYVIR)	Completed	Spain
NCT04758533	Diffuse intrinsic pontine glioma and medulloblastoma	Allogeneic MSC-ICOVIR-5 (AloCELYVIR)	Recruiting	Spain
NCT03896568	Recurrent glioblastoma, gliosarcoma and astrocytoma	Allogeneic MSC-DNX-2401	Recruiting	United States
NCT02068794	Recurrent ovarian, primary peritoneal or fallopian tube cancer	MSC-MV-NIS	Recruiting	United States
NCT02008539	Advanced gastrointestinal adenocarcinoma	Autologous MSC-HSV-TK (MSC_apceth_101) + GCV	Completed	Germany
NCT03298763	Metastatic lung adenocarcinoma	MSC-TRAIL	Recruiting	United Kingdom
NCT02530047	Ovarian cancer	MSC-IFN-β	Completed	United States
NCT02079324	Head and neck cancer	MSC-IL-12 (GX-051)	Unknown status	South Korea
NCT03608631	Metastatic pancreatic ductal adenocarcinoma with KrasG12D mutation	MSC-EV-siRNA KrasG12D (iExosomes)	Active, Not recruiting	United States
